# Assessing the potential of HTA to inform resource allocation decisions in low-income settings: The case of Malawi

**DOI:** 10.3389/fpubh.2022.1010702

**Published:** 2022-10-28

**Authors:** Francesco Ramponi, Pakwanja Twea, Benson Chilima, Dominic Nkhoma, Isabel Kazanga Chiumia, Gerald Manthalu, Joseph Mfutso-Bengo, Paul Revill, Michael Drummond, Mark Sculpher

**Affiliations:** ^1^Centre for Health Economics, University of York, Heslington, United Kingdom; ^2^ISGlobal, Hospital Clínic, Universitat de Barcelona, Barcelona, Spain; ^3^Department of Planning and Policy Development, Ministry of Health Malawi, Lilongwe, Malawi; ^4^Public Health Institute, Ministry of Health Malawi, Lilongwe, Malawi; ^5^Health Economics and Policy Unit (HEPU), College of Medicine, University of Malawi, Zomba, Malawi

**Keywords:** health technology assessment (HTA), resource allocation in health care, healthcare decision making, low- and middle-income countries, Malawi

## Abstract

Health technology assessment (HTA) offers a set of analytical tools to support health systems' decisions about resource allocation. Although there is increasing interest in these tools across the world, including in some middle-income countries, they remain rarely used in low-income countries (LICs). In general, the focus of HTA is narrow, mostly limited to assessments of efficacy and cost-effectiveness. However, the principles of HTA can be used to support a broader series of decisions regarding new health technologies. We examine the potential for this broad use of HTA in LICs, with a focus on Malawi. We develop a framework to classify the main decisions on health technologies within health systems. The framework covers decisions on identifying and prioritizing technologies for detailed assessment, deciding whether to adopt an intervention, assessing alternative investments for implementation and scale-up, and undertaking further research activities. We consider the relevance of the framework to policymakers in Malawi and we use two health technologies as examples to investigate the main barriers and enablers to the use of HTA methods. Although the scarcity of local data, expertise, and other resources could risk limiting the operationalisation of HTA in LICs, we argue that even in highly resource constrained health systems, such as in Malawi, the use of HTA to support a broad range of decisions is feasible and desirable.

## Key contributions

- We illustrate the potential role of research and analysis in informing a broad range of decisions about new health technologies in low-income settings, covering: identifying and prioritizing technologies for appraisal, adoption decisions, implementation and scale-up, and commissioning further research.
- We focus on the context of Malawi, where we use two contrasting technologies (CT scanners and HIV self-testing) as examples to show that an absence of appropriate use of evidence and analysis can lead to decisions that are detrimental to population health.
- We illustrate that it could be feasible to introduce HTA methods also in other highly resource constrained health systems and this could produce tangible results.

## Introduction

### HTA in low-income countries

The challenges of resource allocation and investment decisions in health care are common to all countries. However, governments in low-income countries (LICs), in particular, operate in a context of extreme scarcity of resources and need to make difficult decisions. In such settings there is a high opportunity cost associated with poor decisions: the same funding devoted to a small number of poor value interventions can have larger negative effects on population health than in higher income countries; and missing opportunities to invest in high value technologies results in larger missed opportunities to enhance population health. The fewer the country's resources, the more the need for appropriate evidence and analysis based on the principles of HTA to inform rational decisions on investments, to prioritize needs, and to assess the value of health technologies and their implementation (1). Therefore, in such contexts, there are significant potential efficiency gains from establishing HTA capabilities and strengthening the link between HTA mechanisms and decision-making, thereby enhancing accountability of the technology adoption process (2–4).

While HTA approaches based on evidence and analysis to inform decisions on health technologies are extensively established in high-income countries, these remain weak and underdeveloped in low- and middle-income countries (LMICs), and particularly in LICs. However, in some upper middle-income countries (MICs) some examples of integrated decision-making processes can be found (5, 6). A brief literature review of the current use of HTA in LMICs is reported in Box 1. The key bottlenecks to effective institutional mechanisms for HTA in LICs are threefold. First, the scarcity or lack of financial and human resources for undertaking research and analysis on resource allocation and assessing the quality of HTAs (10, 11, 15, 17, 23). Secondly, there is limited and unreliable locally-relevant data to inform country specific HTAs (24–26). Thirdly, more formal local decision-making procedures, such as the use of HTA that simultaneously considers costs and benefits, are often lacking and there is limited understanding by policymakers of its potential value (9, 13).

Box 1 **Brief literature review of current use of HTA in LMICs.**

**Upper-middle income economies**. In Asia, Thailand has established a semi-autonomous research arm of Ministry of Health (HITAP) which is one of most influential HTA agencies of the region. It uses explicit HTA methods and provides recommendations to assist decision-makers in the prioritisation of health interventions (7). Malaysia uses HTA methods to inform decisions in formulary management; Indonesia for priority setting (8). China uses formal processes to inform decision-making in terms of health insurance benefits and for essential medicine selection and priority settings (9). In Latin America, several upper-MICs conduct HTA-related activities; however, the implementation of HTA remains at a low level and has still a limited formal role in the current legislative framework. Established HTA agencies or committees exist only in countries with more experience in HTA, such as Mexico, Brazil, Argentina, and more recently Colombia. Other upper-MICs such as Costa Rica, Ecuador, Peru, and Venezuela are introducing HTA methods to inform health care decisions (10–12). In Africa, South Africa uses formal processes to inform priority setting and decisions on health insurance benefits and essential medicine selection. The country has committed to strengthening HTA capacity and developing its domestic HTA systems (8, 9); the imminent objective of introducing a national health insurance program provides an opportunity to formally introduce the use of HTA in decision-making (13).

**Lower-middle income economies**. HTA methods are getting more recognition in selected lower-MICs in Africa, such as Egypt (14), Morocco, Tunisia, and Cote d'Ivoire (8). In particular, Ghana has undertaken a pilot study to investigate the use of HTA for specific health technologies. HTA methods have been used to guide the national MOH in priority setting, and this helped to make a case for an institutionalised approach to HTA in the country (8, 15, 16). In Asia, a few lower-MICs are drawing from Thailand's experience, such as the Philippines and Vietnam, which are introducing HTA methods in their health system (7). India uses formal processes to inform decision-making in terms of health insurance benefits (9). Some evidence of informal use of HTA exists also in Myanmar (7).

**Low-income economies**. One of the few LICs which established an HTA committee and is exploring the use of HTA principles to revise the Essential Medicine List is Tanzania (17, 18). Similarly, in Uganda, a HTA association has been formed, with the objective of integrating HTA in the health system (19). Ethiopia is also preparing the implementation of various reforms to improve access to health services, and recently established a team in the MOH, which is mandated to generate high-level evidence to support health policy decision-making and to institutionalise the current fragmented HTA-related activities (20). Local decision-makers in Nepal are showing more recognition of HTA approaches to inform priority setting (8), guide revisions to the country's Essential Medicine List, and expand access to essential health services for the population (21). In Afghanistan and Rwanda, HTA methods are sometimes used informally; no evidence of formal HTA performed through dedicated, independent bodies was found (22). These countries are, however, far from having HTA approaches intergrated routinely in their health systems and decision-making processes.

### The need and the potential for HTA in Malawi

This paper explores the role for HTA in low-income countries with a particular focus on Malawi, a country with one of the lowest GDP per capita in the world. Resources available for health care are particularly scarce, amounting to only $39.5 per person annually (27). Since 2004, Malawi has implemented an Essential Health Package (EHP) to guide investments in health interventions that can be expected to lead to net gains in population health (28). The EHP includes mainly community, primary, and secondary health care services and is revised only every 5 years. Therefore, some technologies which become available in the period between revisions are not considered for the EHP, but the Ministry of Health (MOH) may nevertheless commit resources toward their funding. For example, emerging burden of disease data on non-communicable diseases have recently become available, together with evidence on the increasing cost of foreign referrals for cancer cases. This has motivated the MOH to consider investments in tertiary services and technologies for interventions that are outside the EHP, such as cancer treatments, to tackle this increasing burden to reduce the external referral budget.

These potential investments in new health technologies are currently typically identified through a range of internal processes, with clinical specialists, patient groups or other stakeholders advocating for the adoption of the technology in their facility; or externally, with manufacturers or donors proposing the adoption of new pharmaceuticals, therapeutic or diagnostic equipment. Because the Malawi government has limited funding for new technologies and is not regarded as a major market, the direct influence of manufacturers at country level is limited. However, it is sometimes believed that commercial interests play out indirectly, with manufacturers lobbying for new investments by influencing specialist clinical guidelines or donors, who have the ability to offer funding or co-funding for new services or interventions. For example, during the COVID pandemic, influenced by manufacturers, donors have approached the Ministry to propose the adoption of oxygen-related innovations to treat COVID patients.

The MOH has established technical working groups (TWGs), comprising of ministry officials and other partners, that deliberate policy changes informed by research or analysis. Their recommendations are then passed to MOH management for decision-making. However, in practice, Malawi lacks a structured, systematic, rational, and transparent approach to address the range of decisions associated with new technologies in a consistent manner. This is because of constraints on both the assessment component of HTA (i.e., analysis and research), such as lack of financial resources, scarcity of human resources with appropriate expertise and time to devote to these activities; and the appraisal component (i.e., decision-making), mainly due to decision-makers' lack of awareness about HTA approaches and the political pressures they are under. Decision-making in the health system is thus frequently driven by informal processes, with a lack of predictability about how decisions will be made.

As a result, without a formal structure, the MOH follows a largely unstructured approach when choosing which medicines to buy among the National Essential Medicines List (29), as the listed interventions cannot be fully provided to the population due to lack of resources. Similarly, it is not uncommon for the MOH to be pressured by political or economic interests to procure medical diagnostics, and to follow a series of *ad hoc* approaches, including appealing to the “rule of rescue” given the large number of desperate health needs in the country, rather than to adopt approaches that are based upon a system-wide perspective and could generate larger health gains for the whole population given the budget constraints faced. For example, the MOH procured CT scanners and mammography machines, despite stakeholders deeming such investments as costly in the context of Malawi's health financing. Further examples of technologies that have been adopted (often under donor influence) without formal HTA include vaccines for malaria, oral cholera, and HPV. Many of these adoption decisions may have been appropriate. However, for example, when female condoms were proposed for introduction by donors to promote gender equality, there was no consideration for potential lack of acceptability among the users in Malawi, resulting in limited success. These examples, as well as an expressed need by local policymakers, suggest that establishing an appropriate process locally that considers evidence relating to the context could improve adoption decisions.

### Objectives of this work

Health systems face a broad range of policy decisions related to new technologies. These decisions relate to the different stages through which a technology must pass, from being identified as a potentially viable option within a health care system, to being implemented at scale to provide the greatest impact on population health net of the opportunity costs associated with its funding. The types of evidence and analysis needed to support the different policy decisions are central to HTA, but typically the focus has been on research to support adoption (i.e., whether to fund an intervention and for whom) (30, 31). However, the extensive methods toolbox that has been developed for HTA can and should inform the whole range of policy decisions. Our aim was to develop and describe a framework that classifies these main policy decisions on new health technologies and illustrate, using real examples, how it can be applied in the context of Malawi's health system.

## The use of evidence and analysis: Getting from identification to implementation

Building upon previous examples [e.g. Tugwell et al. (32) and Goodman (33)] our framework illustrates how HTA methods can inform decision-making and guide health spending in LICs. We define four main stages, corresponding to when resource allocation decisions need to be made: (i) “identification and prioritisation” of technologies for assessment; (ii) “adoption” decisions, as to whether particular technologies should be funded within the health system; (iii) “implementation and scale-up” of technologies for widespread use; and (iv) “further research initiatives” that may be valuable in reducing uncertainties relating to these decisions. For each stage, we identify the policy decision to address; illustrate how evidence and analysis can support each decision; and show how an explicit, transparent and systematic approach to funding could be adopted. The order of the stages is not fixed and iterating between them will sometimes be necessary. The full framework is summarized in Table 1. Below we illustrate each stage in turn.

**Table 1:** Framework to classify the main policy decisions on health technologies.

**Stage**	**Policy decision**	**Evidence and methods**
1. Identification and prioritisation	What technologies are potentially valuable to the health system and warrant more detailed assessment?	• Horizon scanning methods • Assess implications of any HTA studies undertaken outside jurisdiction • Consideration of high priority disease areas in jurisdiction (e.g., based on disease burden, high unmet need, policy objectives) • Assess likelihood that manufacturers of proprietary technologies will be flexible in pricing for low-income settings • Assessment of funding sources and opportunity costs
2. Adoption	Does the technology offer value—i.e., does it offer greater additional benefits over existing “comparator” interventions, given the system's objectives, compared to the benefits the same resources could generate elsewhere in the system?	• Systematic review • Evidence synthesis • Decision analytic modelling • Economic evaluation (e.g., CEA) • Assessment of funding sources and opportunity costs
3. Implementation and scale-up	Are adopted technologies available and used as indicated in the adoption decision (appropriate centres, clinicians, patients)? If not, what are the barriers to this and what investments to enhance implementation are of value?	• Qualitative research into barriers to implementation and update • Systematic reviews, synthesis and modelling relating to implementation interventions to overcome barriers • Economic valuation (e.g., value of implementation analysis) • Assessment of funding sources and opportunity costs
4. Further research initiatives	Given uncertainty relating to the above decisions due to limitations in existing evidence (e.g., relevance, quality, precision), does investment in additional studies and data collection offer value?	• Assessment of feasibility of research (e.g., pilots, ethics, qualitative research) • Consideration of appropriate research designs with their associated costs and time to reporting • Economic evaluation (e.g., value of information analysis) • Assessment of funding sources and opportunity costs

### Identification and prioritization

Globally, formal use of evidence and analysis to identify and prioritize technologies for potential adoption and use are mostly not present (34, 35). In LICs, new technologies are often considered for funding for a number of reasons, including recommendations by international agencies (such as the World Health Organization), preferences of donor organizations, advice from specialist clinicians, or pressure being applied by manufacturers or patient organizations (5). Health systems often lack the capacity to evaluate all potentially fundable health care interventions. HTA methods could thus provide a structured, explicit and systematic approach to the identification of health technologies for evaluation. Horizon scanning procedures, which identify those technologies for which there will be potential benefit from uptake or external pressure to use in the near future, together with early-stage assessments based on explicit criteria, could be used, perhaps in partnership with other countries in the region with similar health needs (3, 5, 35, 36). In addition to cushioning decision-makers from external pressures, such approaches could improve transparency of the identification process and expand the consideration set of potentially beneficial technologies.

### Adoption

To establish whether the technology is sufficiently valuable to be adopted (i.e., whether to fund it and for whom), it is necessary to define the criteria for selection and for these to reflect the health system's objectives, taking into account culture, social values, and institutional context of the country. These are typically centered around social values of efficiency and equity (37, 38).

As a key component of HTA, cost-effectiveness analysis (CEA) requires the estimation of incremental cost and health benefits of the new technology compared with existing forms of management. The resulting incremental cost-effectiveness ratio can then be compared with a threshold value of a health outcome, such as a disability-adjusted life-year (DALY), which represents the rate at which a healthcare decision-maker is willing to substitute health outcomes and resources. Because the health care budget is fixed, the value of the threshold should be based on the estimate of the opportunity cost of health care expenditure; that is, the health forgone in the health system when displacing resources elsewhere for the adoption of a new technology. Most recent estimates of the cost-effectiveness threshold for Malawi, based on opportunity cost, are of US$3-116 per DALY averted (39).

Analytical methods exist to support decisions using a wider set of objectives than health maximization. For example, multi-criteria decision analysis (MCDA) has been advocated and used in LMICs (8), but this method has generally not explicitly considered opportunity costs (40). Distributional CEA has been used to assess the value of interventions when policy objectives relate to both gains in aggregate population health and reductions in health inequalities (41, 42). However, whatever the form of economic analysis undertaken, there is a need for it to reflect the relevant clinical, epidemiological and economic evidence. Systematic approaches for identifying such evidence are key to any HTA method (43).

For the analysis of evidence, a summary estimate of clinical effect can be obtained using meta-analysis techniques, and decision-analytic models can be used to synthesize a broad range of clinical and economic evidence (44). However, evidence relating to new interventions is generated worldwide, while decisions are made locally. There is therefore always a challenge to assess the relevance of international evidence to jurisdiction-specific policy decisions: in some cases, evidence will be considered generalisable, in other cases not (45). This is particularly problematic for low-income countries with limited local evidence, and has implications for the uncertainty facing decision-makers.

### Implementation and scale-up

An adoption decision to fund a new cost-effective technology does not guarantee that the technology would be similarly cost-effective during implementation. Implementation setting could differ from that where the results of the analysis relate to. Therefore, if adopted, the health system has to ensure the technology is actually made available to patients and being used appropriately. For this, quality control, monitoring and auditing play a key role. It could be found that uptake of the technology is limited, requiring further investigation of possible barriers to change. A range of decisions might stem from this which can be informed by analysis consistent with the principles of HTA. Methods can be used to take into account local conditions and resource input constraints (e.g., shortage of skilled labor) to optimize implementation and scale up (46, 47). Value of implementation analysis can inform policy decisions about investments used to overcome identified barriers (i.e., implementation strategies such as training, health system changes, guidelines) (48, 49).

### Further research initiatives

Typically, the use of HTA methods to inform decisions initially relies on synthesis of existing (secondary) evidence and models to apply the evidence to the specified decision problems. However, published studies may be of doubtful relevance to the local setting, dated or of low quality, and evidence on key effects of new interventions may be lacking. Policy decisions are hence likely to be taken under uncertainty, and this may be particularly extreme in LICs. The inevitability of uncertainty in decisions raises the prospect of a further type of decision—the investment in additional research with local relevance, to generate new evidence.

Research requires resources that could have been devoted to health care, so it imposes opportunity costs in terms of health outcomes. However, research could for example help to acquire more information to reduce uncertainties and inform potential resource constraints on expenditure, thus facilitating better decisions, with positive implications for population health. Value of information (VOI) analysis can provide a formal assessment of the cost that health systems incur in making decisions under uncertainty in terms of population health and resources, the implied value of additional research and appropriate design of research studies (50). VOI methods can also inform the adoption decision—for example, by indicating whether additional research should come before possible adoption of a technology or alongside its general diffusion (51). In LICs, research could inform the potential inclusion of a new technology in an Essential Health Package.

### The importance of considering the channels of funding at each stage

Even though affordability is being considered more and more when assessing technologies in LMICs, the decision on how to finance new technologies is typically not explicitly considered in HTA in high-income settings. There is generally an implicit assumption that any additional financial resources will be generated through additional taxation or insurance premiums, or that the system will find the funding from its existing budget by reducing, or disinvesting from, existing activities, although these sources of funding are rarely explicitly identified or verified. Furthermore, the implications for funding extend beyond the adoption decision (where HTA tends to focus) and include the cost of scale up and appropriate update and use. The framework described here suggests the potential for funding should be considered at an early stage, since particularly in LICs it is not worth investing scarce resources in a technology for which affordability issues would prevent adoption.

If not addressed in a systematic fashion, funding decisions may only take into consideration very specific cost components and ignore the broad spectrum of costs. Further, the existence of different sources of funding might be overlooked. Particularly in LICs, resources may be provided by external donor agencies, in which case it is important to establish whether these are restricted to the initial investment or also include recurring costs. As resources displaced from different public and private budgets are associated with different opportunity costs (i.e., the benefits associated with the alternative use of the same resources), a systematic and explicit approach to funding can help to identify the sources of funding, and correctly reflect the opportunity costs.

## Framework applied in the context of Malawi's health system

### Identification and prioritization

For Malawi, the most rigorous process for prioritization of interventions relates to the EHP. Given the extremely low per capita healthcare spending, effective HTA processes could rationalize expenditures on technologies addressing the most pressing health care needs more cost-effectively (36). A more relevant approach to identify technologies for detailed assessment would be to consider good-quality clinical and economic studies from other countries—ideally middle and low-income settings. Candidate technologies would be those showing significant additional clinical benefits over existing interventions of relevance to Malawi, in priority diseases areas and with incremental costs per unit of health effect that are potentially affordable. Additional considerations might include whether, for proprietary technologies, manufacturers have shown willingness to accept lower prices in low-income countries and whether the technologies have potential to reduce the use of resources for which there are particular shortages in Malawi (e.g., skilled staff or hospital beds).

Since Malawi's health care system is funded through many different sources, a systematic financial analysis could inform decision-makers at this early prioritization stage (5). Of a total annual health expenditure of US$694 in financial year 2017/18, donors and government spent 58.6% and 23.9%, respectively, indicating high reliance of the health system on donor funding. Despite the predominance of donor financing, government is the main agent through which donor funds are channeled, managing at least 48% of total health financing, compared to donors and NGOs who managed 34.7% between 2015/16 and 2017/18 (27, 52). Systematic financial analyses could, firstly, consider the financial and non-financial resources needed for all the stages of the potential introduction of the health technology in the health system. Secondly, it could help to assess the magnitude of health care resources that should be displaced elsewhere to cover new investments, for both Government and donor funding pools, thereby facilitating consideration of the opportunity costs of the resources displaced.

### Adoption

The criteria that have been deemed relevant to decision-makers in Malawi are: gains in population health, equity (e.g., targeting marginalized or rural populations, or women and children under five), existence of complementarities between interventions due to the potential for efficiency savings, and the magnitude of donor funding for the interventions (28). However, Malawi faces a scarcity of local evidence to inform such criteria, and limited capacity and expertise to analyse and interpret the evidence, when available. Further, evidence and analyses are very rarely transferred from other jurisdictions. However, rapid reviews and assessments of comparative safety and efficacy could be feasible approaches that can still produce an early positive impact on decision-making (5).

In the country, all technologies would need to be approved by the Ministry of Health in order to be introduced at facility level. However, although CEA is a prerequisite for public–private partnerships which need Cabinet approval, decisions at national level are largely not systematically informed by considerations of effects and costs, and mostly undertaken through an opinion and experience-based process. To promote transparency and accountability, and to reduce the risk of sub-optimal decisions, policymakers could use more structured and evidence-informed deliberative processes when a range of objectives are relevant to their decisions (53).

### Implementation and scale-up

In Malawi, local conditions and constraints influence the performance of technologies in routine use. For example, considerable input constraints exist in terms of gaps in human resource capacity, with high vacancy rates across human resources for health cadres in the Malawian public health sector, the suboptimal distribution of existing staff and the limited availability and coordination for high quality training. Nevertheless, in the country, most medical technologies are immediately rolled out and only smaller technologies (e.g., rapid tests for HIV, syphilis, and tuberculosis) have been piloted and evaluated before full roll out, although these pilots have been modest in scale and scope.

Sometimes implementation costs are covered by donor funds; for example, for the roll-out of the HPV vaccine, GAVI provided the roll out costs apart from the procurement of the vaccine itself. However, when new technologies are donated or procured with donated funds, budgets for service and related costs are rarely included. Whilst central hospitals have always had discretion in the allocation of resources, including maintenance, since 2005 district maintenance budgets have been decentralized. Districts take care of operational costs associated with using the technologies, and sections of the health service are expected to fund the growing use of a technology from their existing budgets. Some maintenance budget is at the headquarters level which is used to support both districts and central hospitals. The implementation of new interventions can also be highly dependent on the views, perceptions, and interests of key stakeholders. For example, when female condoms were introduced in Malawi, low acceptability of the intervention by the local population played a crucial role in the low uptake of the intervention.

To monitor technologies' roll-out and use in regular practice in Malawi, a Health Management Information System (HMIS), which includes the District Health Information Software (DHIS2), is in place for data collection and reporting. In principle, this could provide information on new technologies in the system. However, the HMIS still has many weaknesses related to poor data quality, and parallel reporting systems such as programme-specific monitoring and evaluation frameworks. Furthermore, it relies on manual data collection and reporting processes. At each level of the health system (district, central hospital and headquarters), regular audits should be conducted by the Internal Audit Units. However, insufficient funding to conduct field visits and inadequate capacity limit their capability to prepare reports for the Independent Audit Committee for Health (28).

The evaluation system in Malawi is weaker than the monitoring one. A Quality Management Department has been established by the MOH with the aim of improving on health care quality. Initiatives are in place to ensure that each facility has quality assurance teams ensuring standards of quality are met. However, quality control testing by the National Quality Control Laboratory to assess the safety and efficacy of medicines is limited, and the capacity of Drug and Therapeutic Committees in most health facilities is weak. National accreditation standards are under development. It has been reported that quantity and quality of health care equipment is low and not routinely tracked, and almost 25% is out of service (28).

### Further research initiatives

The Malawian MOH does not have a specific systematic process to determine the types of research that are most needed. Research is conducted in an *ad hoc* manner, and the National Health Research Agenda does not necessarily reflect what research has the greatest potential to improve population health in the country. When conducted, additional research is typically supported by external sources of funding. The MOH does not have a national budget for health care research on the costs and benefits of new technologies (54), but there is a recommendation to allocate 2% of the health budget toward research (55). However, because there has not been a process in place to assess and prioritize research needs, this funding has remained uncommitted. The Health Services Joint Fund (56), co-financed by the governments of the UK, Norway and Germany, could act as a catalyst to attract resources and funding for research. However, weak institutional mechanisms to support research decisions in the health care sector could partly explain the low funding for research.

### Framework applied to two health technologies in Malawi

To illustrate the current use of HTA methods in Malawi and their potential to inform decisions, we present two technologies as examples. In Box 2, we describe how decisions were addressed when introducing CT scanners in the Malawian health system. In Box 3, we show mechanisms by which evidence can enter policy deliberations using HIV self-testing as an example.

Box 2 **CT scanners (an actual decision already taken).**

In recent years, the MOH in Malawi has faced pressures to acquire CT scanners from clinicians, who argued that the available scanners were old and had been purchased second hand, and from patients, who did not want to pay for the tests in private hospitals. The technology was hence considered for adoption only because of political pressures, and not because it was identified through a formal assessment process.

When deciding about their adoption, the MOH did not assess the priority of introducing CT scanners compared with funding other technologies or other potential uses of limited budgets. Furthermore, it did not investigate the available purchasing and use options before making a decision. Questions on cost-effectiveness and value for money were raised, but there was no expertise or even time, given the political economy pressures, to address the problem with an economic evaluation.

As a result, in 2018 the MOH acquired two CT scanners; one for the Queen Elizabeth Central Hospital, which due to delayed procurement only became operational in 2020, and the other as a repair of the existing one at Kamuzu Central Hospital. To cover the initial purchase, as well as for user training and services contracts, the MOH received funds from Norwegian Development Aid through the Health Services Joint Fund (57). Other implementation costs, such as consumables and operating supplies were to be covered under the applicable hospital budget, which means less funding is consequentially available for other services. To start operating the machines, scanners were assessed by the Health Technical Support Services Department at the MOH. Routine monitoring was planned, and no decisions on further research on the use of the scanners were taken.

In principle, a process would have ideally already been in place in the MOH to commission research teams to investigate the evidence, and present their findings to a TWG, which then gives its recommendation to MOH senior management. However, due to time and resources constraints, evidence is formally assessed only in some specific cases, such as COVID vaccines. Nevertheless, even if it was not feasible to conduct a context-specific economic evaluation due to such constraints, a quick review of the evidence would have helped to protect decision-makers from external pressures pushing for the adoption of the scanners. In fact, even if locally relevant evidence is not available, evidence from the U.S. shows that CT screening was associated with an ICER of US$151,000 per life-year gained, when compared with routine care (58). There are reasons to believe that, in the context of Malawi, benefits of the technology are likely to be smaller. This is due to a low level of integration of imaging into the entire health service delivery, limited human resource with the required skills, insufficient awareness for radiation safety awareness, insufficient facilities and opportunities for education and training (59). Costs of the equipment are likely to be transferrable, whereas the generalisability of costs of disease management and follow-up testing should be investigated in detail. However, it is possible that costs may be higher due to a scarcity of qualified engineers to conduct maintenance, import costs, and additional potential requirements (e.g., generator) (60). Therefore, the value of the ICER associated with CT scanner in Malawi would probably be even higher than what estimated has been estimated for the US. This value is clearly well beyond the supply-side cost-effectiveness threshold estimates for Malawi of US$3-116 per DALY averted (39). On the basis of such evidence, it could have been possible to formally assess whether it would have been preferable to leave this technology available only in private hospitals, with co-financing, instead of adopting it as a publicly funded service.

Box 3 **HIV self-testing (a decision that has yet to be taken).**

HIV self-testing is in use in the country, but we observe a limited commitment of public money to support its use, which mainly relies on donor funding. The technology has not been assessed through a formal process by the Malawi government, and its scaling up is slow (61). In such a context, a transparent process informed by analysis and evidence could help to inform a wide range of decisions in an objective manner.

The MOH could establish a horizon scanning process based on the experience of other countries, or through a partnership with other countries, and a prioritisation mechanism based on factors such as burden of disease, unmet need, potential for cost-saving, public interest, or availability of scientific evidence. If so, HIV self-testing would likely look promising for widespread adoption as it is an effective and low-cost technology that has been demonstrated the potential to provide health benefits and save health care resources in LICs (62). On the basis of this evidence, the Department of Planning and Policy Development, or the Health Technical Support Services and its sub-departments (e.g., Diagnostics, Pharmaceuticals, and Physical assets management) could conduct a preliminary budget impact assessment, and possibly consider whether donor funding could cover acquisition costs.

Clinical and economic evidence relevant to Malawi is available and indicates that HIV self-testing may serve to overcome barriers of current testing models in Sub-Saharan Africa (63) and has the potential of improving men's uptake in HIV testing services (64). Evidence confirms high feasibility, acceptability and accuracy across many delivery models and populations in the region (65). Facility-based HIV self-testing increased HIV testing among outpatients in Malawi (61).

In terms of cost-effectiveness, evidence shows that the introduction of self-testing in Zimbabwe would allow savings of around $75 million over 20 years and approximately 7,000 DALYs averted. If such evidence is generalisable to the context of Malawi, the technology should be recommended as it generates better health outcomes at reduced cost (62). Additional evidence indicates that community-based HIV self-testing is generally cost-effective (i.e. ICER below US$500 per DALY averted) if introduced in sub-Saharan Africa in context where prevalence of undiagnosed HIV among adult men is sufficiently high, or targeted to women having transactional sex. Further, to provide more accurate recommendations for the Malawian setting, cost-per-diagnosis can be used to monitor the cost-effectiveness of testing programmes (66, 67). However, cost-effectiveness may not be the solely relevant criteria to decide about the adoption of the technology. Therefore, a structured and transparent evidence-informed decision process could be followed, based on explicitly stated decision criteria that take into consideration institutional context and values, and social and political sensitivity.

If and when adopted, further research commissioned by the TWGs could inform the inclusion of the HIV self-testing in the Essential Health Package. However, further analyses would be required to investigate whether scaling-up would be feasible, given the resource constraints challenges (61).

## Discussion

HTA methods based on evidence and analysis have significant potential to support a wide range of decisions related to the potential introduction of health technologies and their subsequent delivery in the health systems of LICs. With this work, we argue that the proposed framework can be adopted in Malawi, with methods that are feasible given the resource and capacity constraints faced. The framework can also be used when attempting to introduce HTA into other LICs that have similar health systems. However, we recommend that the framework is discussed in further consultation with a wide array of stakeholders in Malawi and beyond, such as patients and community representatives, professional bodies, non-governmental organizations and international health organizations, for further refinements before its adoption.

The benefits of introducing HTA processes in Malawi are numerous. Most obviously, HTA could lead to better use of limited resources by guiding investments in technologies that are more likely to be of value in Malawi's national context. HTA is not intended to block the availability of particular interventions; if these do not represent a valuable use of limited health care budgets in the public sector, they could still be funded out of other sources (e.g., private payments) where information from HTA on the health effects for individual patients is important for consumers' decisions. The use of HTA may also have a wide range of additional benefits. For example, it may help to align various stakeholders around national health system objectives. Malawi, like many LICs, is heavily reliant on external funding from donors to its health system. By illustrating trade-offs and making comparisons across different technologies and calls upon limited funds, HTA has the potential to facilitate government and donors working together more effectively in meeting the health needs of the population. It could avoid, for instance, situations where interventions are adopted only because they are recommended by international agencies without sufficient recognition of within-country resource constraints and opportunity costs. Where particular interventions are too expensive for Malawi, the evidence may also be used in strengthening price negotiations (21), especially if such action is coordinated across a range of similar countries (for instance, in the East Central and Southern Africa Health Community, ECSA-HC).

Effective partnerships between researchers, HTA specialists and key government stakeholders (e.g., government officials from the planning and policy development, public health and clinical departments) are crucial to ensure that HTA is a success in terms of research and analysis informing real decision-making in a timely manner. To foster its institutionalization, HTA needs to be endorsed by senior decision-makers as well as the providers of evidence. An initial goal for any LIC could be to familiarize decision-makers and technical officers to HTA principles and practices, to promote stakeholder engagement (7, 9). Moreover, HTA decision-making powers are currently spread across the Malawian MOH. Therefore, a more ambitious objective could be the creation of a HTA committee, as attempted in Tanzania (17). This could help to limit the uncoordinated approach of adopting measures, and facilitate the collaboration and cooperation between government authorities with related, but differing mandates, for instance departments of ministries of health and ministries of finance, as well as other stakeholders (13).

The decision for how HTA is established in Malawi is one for the MOH Management team, comprising relevant MOH Departments, with particular support likely to emerge from the MOH Department of Planning and Policy Development. MOH would be supported by existing relevant TWGs, such as the Health Services TWG, or a new TWG for HTA could be started. Moreover, there is likely to be need for important roles for the academic sector in Malawi and use of existing research-to-policy channels. There is therefore scope for institutions such as the Health Economics and Policy Unit (HEPU), housed by Kamuzu University of Health Sciences, with its think tank and policy lab to present evidence to policymakers, in order for it to be considered in policy deliberations. Successful synergies between HEPU and MOH, already permitted the use of evidence from other countries to improve the identification strategies of COVID-19 cases. Furthermore, the MOH Research Division and the Malawi Knowledge Translation Platform, which aim to translate scientific language to policy language to inform senior management, could help to link research institutions with policymaking reality.

Establishing prioritization systems in LICs and operationalizing HTA may be hampered by the scarcity of resources, capacity and data. However, health economic evidence has grown substantially in most Sub-Saharan African countries and is becoming more easily accessible thanks to initiatives such as the Disease Control Priorities (68) and TUFTS Global Health Cost-Effectiveness Registry (69). However, the capacity for generating such evidence and putting it into use within local institutions remains limited (24). In LMICs it is thus commonplace to transfer evidence and analyses from other jurisdictions, even though such evidence is sometimes challenging to use, due to differences for example in standard of care or prices between jurisdictions (45). However, this opportunity is very rarely utilized in LICs, such as Tanzania (18). Further, when evidence is available, LICs typically face limited capacity and limited expertise to analyse and interpret the evidence, as shown for example in Ethiopia (20). However, for countries with very limited HTA capacity such as Malawi and most LICs, feasible approaches exists, such as rapid reviews and assessments of comparative safety and efficacy (5), until within-country capacity is further developed.

International collaboration and capability building can also help, accompanied by shared analytical capacity, resources and expertise among LMICs can help to overcome the resourcing limits for HTA in LICs, and strengthen individual, institutional and organizational capacity (4, 17, 70). The development of regional analytical capacity through intergovernmental agencies, such as ECSA-HC, can also be a key factor for the development of HTA processes. Because the existence of local capacity is a crucial conducive factor for the development of HTA approaches (21), international support should focus on extending and further developing evidence databases, promoting capability building and generating expertise on how to use HTA in decision-making.

## Data availability statement

The original contributions presented in the study are included in the article/supplementary material, further inquiries can be directed to the corresponding author.

## Author contributions

PR, MD, MS, and JM-B conceived the study. FR wrote the first draft of the manuscript with inputs from all authors. PT, BC, DN, IK, GM, PR, MD, MS, and JM-B provided critical revision with substantial intellectual contributions. FR wrote the final version of the article. All authors reviewed and approved the final manuscript.

## Funding

This study was funded by UK Research and Innovation as part of the Global Challenges Research Fund (Thanzi la Onse grant number MR/P028004/1).

## Conflict of interest

The authors declare that the research was conducted in the absence of any commercial or financial relationships that could be construed as a potential conflict of interest.

## Publisher's note

All claims expressed in this article are solely those of the authors and do not necessarily represent those of their affiliated organizations, or those of the publisher, the editors and the reviewers. Any product that may be evaluated in this article, or claim that may be made by its manufacturer, is not guaranteed or endorsed by the publisher.
